# The role of metabolism in bacterial persistence

**DOI:** 10.3389/fmicb.2014.00070

**Published:** 2014-03-03

**Authors:** Stephanie M. Amato, Christopher H. Fazen, Theresa C. Henry, Wendy W. K. Mok, Mehmet A. Orman, Elizabeth L. Sandvik, Katherine G. Volzing, Mark P. Brynildsen

**Affiliations:** ^1^Department of Chemical and Biological Engineering, Princeton UniversityPrinceton, NJ, USA; ^2^Department of Molecular Biology, Princeton UniversityPrinceton, NJ, USA; ^3^Rutgers Robert Wood Johnson Medical School, Rutgers UniversityPiscataway, NJ, USA

**Keywords:** bacterial persistence, metabolism, antibiotic tolerance, ppGpp, nutrient environment

## Abstract

Bacterial persisters are phenotypic variants with extraordinary tolerances toward antibiotics. Persister survival has been attributed to inhibition of essential cell functions during antibiotic stress, followed by reversal of the process and resumption of growth upon removal of the antibiotic. Metabolism plays a critical role in this process, since it participates in the entry, maintenance, and exit from the persister phenotype. Here, we review the experimental evidence that demonstrates the importance of metabolism to persistence, highlight the successes and potential of targeting metabolism in the search for anti-persister therapies, and discuss the current methods and challenges to understand persister physiology.

## Introduction

**Figure 1:**
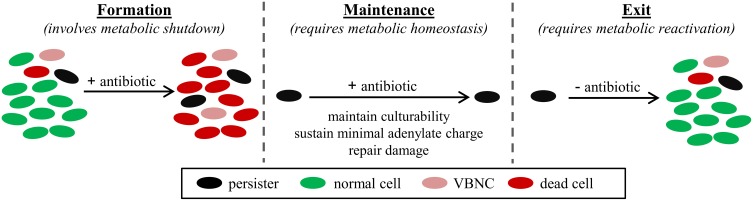
**Persister metabolic program**. Persisters can be pre-existing in a bacterial population (Balaban et al., 2004), formed in response to stress, such as stresses that activate the stringent response (Amato et al., 2013), and induced during antibiotic treatment (Dörr et al., 2009; Orman and Brynildsen, 2013a). Maintenance of the persister state for the duration of the antibiotic treatment requires temporary inhibition of essential cell functions; however, persisters must remain culturable, which requires a minimal adenylate charge to be sustained (Chapman et al., 1971) and damage to be repaired (Nystrom and Gustavsson, 1998). Upon removal of the antibiotic, persisters exit their tolerant state and give rise to a bacterial population of identical antibiotic susceptibility as the original population (Balaban et al., 2004).

Bacterial cultures contain a small subpopulation of cells that cannot readily be killed by antibiotics (Bigger, 1944). These cells have been named persisters, and their existence can be detected from antibiotic kill curves, where the first, rapid killing regime represents the death of normal cells and the second, slower killing regime indicates the presence of persisters (Balaban et al., 2004; Kint et al., 2012). Further, when these survivors are cultured, they produce populations with antibiotic sensitivities identical to those of the original culture. This establishes persistence as a phenotypic trait, unique from antibiotic resistance where genetic determinants allow growth at higher antibiotic concentrations. Persisters are an important health concern because they are enriched in biofilms and thought to underlie the proclivity of biofilm infections to relapse following the conclusion of antibiotic therapy (Lewis, 2008, 2010). Persisters have proven to be difficult to analyze due to their transient nature, low abundance, and similarity to the viable but non-culturable (VBNC) cell-type (Roostalu et al., 2008; Orman and Brynildsen, 2013b). However, strong evidence, in the form of genetic- and microscopy-based data (Balaban et al., 2004; Lewis, 2010; Maisonneuve et al., 2013), exists to support that, while under antibiotic stress, persister tolerances are derived from inactivity of essential cell functions. While this is not always the case, as demonstrated in a study of isoniazid (a prodrug requiring activation) (Wakamoto et al., 2013), and dormancy is not essential for persistence prior to antibiotic stress (Orman and Brynildsen, 2013a), prolonged survival to the majority of antibiotics, in the absence of genetic mutations, requires inactivity of the antibiotic's primary target. To achieve and maintain this state, as well as reverse the process to repopulate environments, coordinated metabolic action is needed. Namely, metabolism would participate in cessation of essential functions, be needed to maintain culturability (e.g., sustain a minimal adenylate charge: [ATP + 0.5ADP]/[ATP + ADP + AMP] (Chapman et al., 1971), repair/resynthesize damaged proteins (Nystrom and Gustavsson, 1998)), and reactivate the cell during reawakening (Figure 1). We refer to this process as the persister metabolic program and summarize the accumulated evidence substantiating the importance of metabolism to the persister phenotype as well as current methods and challenges to studying the metabolism of these rare and transient phenotypic variants.

## Genomic studies identify metabolic genes as important to the persister phenotype

Perturbations to genes that encode enzymes or regulators of metabolism have frequently been found to alter persister levels (Table 1). In one of the initial genomic screens for persistence, a library was generated through digestion of the *Escherichia coli* chromosome, ligation of the fragments into plasmids, and transformation of the library into *E. coli* (Spoering et al., 2006). Upon successive rounds of ampicillin (AMP) treatment and culturing of survivors, a plasmid carrying *glpD*, encoding G3P-dehydrogenase that converts glycerol-3-phosphate (G3P) to dihydroxyacetone-phosphate (DHAP), was found to increase the abundance of persisters. Further analysis identified additional enzymes in G3P metabolism important for persistence to AMP, ofloxacin (OFL), and ciprofloxacin (CIP) (Table 1). The importance of G3P to *E. coli* persistence was further supported by a transposon mutant screen where *glpD* mutants were found to increase persistence after successive rounds of selection on LB-AMP agar (Girgis et al., 2012). This effect was attributed to elevated levels of methylglyoxal, a toxic compound derived from DHAP. Interestingly, these observations, where GlpD inactivation increased persistence, were opposite to those of Spoering and colleagues. However, we note that G3P is a highly connected metabolite, given its proximity to central metabolism, interaction with the quinone pool, and use as a precursor for phospholipid biosynthesis. Therefore, different assay conditions may explain the variable impacts on persistence.

**Table 1:** **Metabolism-associated genes identified through genomic studies to influence persistence**.

**Organism**	**Gene^α^**	**Gene function**	**Metabolic role**	**Mutation**	**Antibiotic^β^**	**Culture conditions^γ^**	**Persistence outcome**	**References**
*E. coli*	*argE*	Acetylornithine deacetylase	AA metabolism	Mutation	TIC, OFL	Biofilm	Increase	Bernier et al., 2013
*E. coli*	*argH*	Argininosuccinate lyase	AA metabolism	Deletion	TIC, OFL	Biofilm	Increase	Bernier et al., 2013
*E. coli*	*aroE*	Shikimate 5-dehydrogenase	AA metabolism	Mutation	TIC, OFL	Biofilm	Increase	Bernier et al., 2013
*E. coli*	*atpA*	F_o_/F_1_ ATP synthase subunit α	Energy production	Mutation	AMP	Plates	Increase	Girgis et al., 2012
*E. coli*	*atpF*	F_o_/F_1_ ATP synthase subunit B	Energy production	Mutation	AMP	Plates	Increase	Girgis et al., 2012
*E. coli*	*cysD*	Sulfate adenylyltransferase subunit 2	AA metabolism	Deletion	TIC	STAT	Increase	Bernier et al., 2013
				Deletion	TIC, OFL	Biofilm	Increase	Bernier et al., 2013
*E. coli*	*dksA*	Transcription regulation	Stringent response regulation	Deletion	OFL, CIP, STM, AMP	STAT, EXP	Decrease	Hansen et al., 2008
*E. coli*	*galU*	Glucose-1-phosphate uridylyltransferase	Glycogen metabolism	Mutation	AMP	Plates	Increase	Girgis et al., 2012
*E. coli*	*glpABC*	Anaerobic G3P dehydrogenase	G3P metabolism	Deletion	CIP	STAT	Decrease	Spoering et al., 2006
*E. coli*	*glpD*	G3P dehydrogenase	G3P metabolism	Mutation	AMP	Plates	Increase	Girgis et al., 2012
				Deletion	CIP	STAT	Decrease	Spoering et al., 2006
				Over-expression	AMP, OFL	EXP	Increase	Spoering et al., 2006
*E. coli*	*hipA*	Serine/threonine kinase	Stringent response regulation	Mutation	AMP, CYC, PHM	EXP, Plates	Increase	Moyed and Bertrand, 1983
*E. coli*	*hipB*	Transcriptional repressor, antitoxin	Stringent response regulation	Mutation	AMP	Plates	Increase	Girgis et al., 2012
*E. coli*	*hisG*	ATP phosphoribosyl-transferase	AA metabolism	Deletion	TIC, OFL	Biofilm	Increase	Bernier et al., 2013
*E. coli*	*ilvA*	Threonine dehydratase	AA metabolism	Deletion	TIC, OFL	Biofilm	Increase	Bernier et al., 2013
*E. coli*	*ilvC*	Ketol-acid reductoisomerase	AA metabolism	Mutation	TIC, OFL	Biofilm	Increase	Bernier et al., 2013
*E. coli*	*livJ*	Leucine/isoleucine/valine transporter	AA transport	Mutation	AMP	Plates	Increase	Girgis et al., 2012
*E. coli*	*leuB*	3-Isopropylmalate dehydrogenase	AA metabolism	Mutation	TIC, OFL	Biofilm	Increase	Bernier et al., 2013
*E. coli*	*leuC*	Isopropylmalate isomerase large subunit	AA metabolism	Mutation	TIC, OFL	Biofilm	Increase	Bernier et al., 2013
				Deletion	TIC	STAT	Increase	Bernier et al., 2013
				Deletion	TIC, OFL	Biofilm	Increase	Bernier et al., 2013
*E. coli*	*lysA*	Diaminopimelate decarboxylase, PLP-binding	AA metabolism	Deletion	TIC	STAT	Increase	Bernier et al., 2013
				Deletion	TIC, OFL	Biofilm	Increase	Bernier et al., 2013
*E. coli*	*metA*	Homoserine *O*-succinyltransferase	AA metabolism	Deletion	TIC, OFL	Biofilm	Increase	Bernier et al., 2013
*E. coli*	*pheA*	Fused chorismate mutase P/prephenate dehydratase	AA metabolism	Deletion	TIC, OFL	Biofilm	Increase	Bernier et al., 2013
*E. coli*	*phoU*	Pho operon repressor	Phosphate metabolism	Mutation	AMP, NOR, PZA, AMP/GEN	STAT, EXP	Decrease	Li and Zhang, 2007
				Deletion	AMP, NOR	STAT, EXP	Decrease	Li and Zhang, 2007
*E. coli*	*proA*	γ-Glutamyl phosphate reductase	AA metabolism	Mutation	TIC, OFL	Biofilm	Increase	Bernier et al., 2013
*E. coli*	*proC*	Pyrroline-5-carboxylate reductase	AA metabolism	Deletion	TIC, OFL	Biofilm	Increase	Bernier et al., 2013
*E. coli*	*sucB*	Dihydrolipoamide acetyltransferase	Energy production	Deletion	AMP, GEN	STAT, EXP	Decrease	Ma et al., 2010
*E. coli*	*tktA*	Transketolase	Energy production	Mutation	AMP	Plates	Increase	Girgis et al., 2012
*E. coli*	*trpA*	Tryptophan synthase subunit α	AA metabolism	Deletion	TIC	Biofilm	Increase	Bernier et al., 2013
*E. coli*	*tyrA*	Fused chorismate mutase T/prephenate dehydratase	AA metabolism	Deletion	TIC, OFL	Biofilm	Increase	Bernier et al., 2013
*E. coli*	*ubiF*	2-Octaprenyl-3-methyl-6-methoxy-1,4-benzoquinone hydroxylase	Energy production	Deletion	AMP, GEN	STAT, EXP	Decrease	Ma et al., 2010
*E. coli*	*ygfA*	5-Formyltetrahydrofolate cyclo-ligase	Coenzyme biosynthesis	Deletion	OFL, CIP, STM, AMP	STAT, EXP	Decrease	Hansen et al., 2008
*P. aeruginosa*	PA14_13680	Putative short-chain dehydrogenase	Unknown	Mutation	OFL	STAT	Increase	De Groote et al., 2009
*P. aeruginosa*	PA14_17880	Acetyl-CoA acetyltransferase	Fatty acid and phospholipid metabolism	Mutation	OFL	STAT	Decrease	De Groote et al., 2009
*P. aeruginosa*	PA4115	Lysine decarboxylase	AA metabolism	Mutation	CB	EXP, Plates	Increase	Manuel et al., 2010
				Deletion	CB, TIC	EXP	Increase	Manuel et al., 2010
*P. aeruginosa*	*pheA*	Prephenate dehydratase	AA metabolism	Mutation	OFL	STAT	Increase	De Groote et al., 2009
*P. aeruginosa*	*spuC*	Putrescine aminotransferase	Polyamine metabolism	Mutation	OFL	STAT	Decrease	De Groote et al., 2009
*P. aeruginosa*	*ycgM*	Putative fumarylaceto-acetate hydrolase family protein	Secondary metabolite biosynthesis, transport, and catabolism	Mutation	OFL	STAT	Increase	De Groote et al., 2009
*S. mutans*	*fruA*	Fructan hydrolase	Carbohydrate metabolism	Fragment over-expression	OFL	STAT	Decrease	Leung and Lévesque, 2012
*S. mutans*	*pfl*	Pyruvate formate-lyase	Energy production	Fragment over-expression	OFL	STAT	Increase	Leung and Lévesque, 2012
*S. mutans*	*scrA/scrB*	Sucrose-specific IIABC PTS component/Sucrose-6-phosphate hydrolase	Carbohydrate metabolism	Promoter over-expression	OFL	STAT	Increase	Leung and Lévesque, 2012
*S. mutans*	*scrR*	Sucrose-PTS operon repressor	Carbohydrate metabolism	Deletion	OFL	STAT	Increase	Leung and Lévesque, 2012
*S. mutans*	SMU.1278	Putative phosphoglycolate phosphatase	Unknown	Fragment over-expression	OFL	STAT	Increase	Leung and Lévesque, 2012

^α^Genes listed in this table are those associated with metabolism that were found to influence persistence in studies that included a genomic screen. Genes not directly connected to metabolism are not listed here, and metabolic genes identified in non-genomic studies are also not presented. ^β^Antibiotic abbreviations: ampicillin (AMP), carbenicillin (CB), ciprofloxacin (CIP), cycloserine (CYC), gentamicin (GEN), norfloxacin (NOR), ofloxacin (OFL), phosphomycin (PHM), pyrazinamide (PZA), streptomycin (STM), ticarcillin (TIC). ^γ^Culture conditions describe the bacterial growth state at the time of antibiotic exposure: exponential (EXP), stationary (STAT), agar plates (Plates), biofilm.

Beyond G3P, genomic studies have found that mutations perturbing amino acid (AA) metabolism significantly influence persistence (Table 1). Screening of an *E. coli* transposon library for persistence to ticarcillin (TIC) or OFL identified 18 mutants with increased persister levels, and of those, 16 mapped to genes involved in AA biosynthesis (Bernier et al., 2013). *Pseudomonas aeruginosa* screens have also uncovered disruptions in AA metabolism as important to persistence. Mutation of PA4115, a putative lysine decarboxylase, was found to increase persistence to carbenicillin (CB) (Manuel et al., 2010), whereas mutation of *pheA*, which is also involved in AA metabolism, was found to increase persistence to OFL (De Groote et al., 2009). These studies suggest that AA metabolism is a critical mediator of persistence, and as one would expect, the stringent response, a major metabolic regulatory system controlled by ppGpp and its transcriptional partner DksA, also mediates persistence (Korch et al., 2003; Viducic et al., 2006; Fung et al., 2010; Nguyen et al., 2011; Amato et al., 2013; Maisonneuve et al., 2013). This influence was also detected in a screen where Δ*dksA* was found to produce far fewer persisters toward OFL (Hansen et al., 2008).

The third major metabolic system that has been shown to impact persistence is energy metabolism. A screen of an *E. coli* transposon library found that deactivation of *phoU* reduced persistence (Li and Zhang, 2007). PhoU is a negative regulator of the phosphate operon, and its inactivation led to a hyperactive metabolic state. In a screen of the Keio collection for AMP persistence, Δ*sucB* and Δ*ubiF* were found to produce lower persister levels (Ma et al., 2010). SucB participates in the TCA cycle, whereas UbiF is an enzyme in ubiquinone biosynthesis, and deactivation of either of these genes leads to deficient energy production. Interestingly, these studies point to both metabolic hyperactivity and inhibition as methods to reduce persistence. One interpretation of these results could be that metabolic hyperactivity reduces entry into the persister state, whereas inhibition of energy production prevents exit from the phenotype. Regardless, energy generation appears to be a critical process to the persister metabolic program.

Collectively, these studies have provided a wealth of evidence on the importance of metabolism to bacterial persistence, even though they have sampled only a fraction of the mutational landscape. The details of how each genetic perturbation affects entry into, maintenance of, or exit from the persister state largely remains to be elucidated; however, it is clear that G3P, AA metabolism, and energy production are all important to defining persistence in a bacterial population.

## Persister levels depend on the nutritional environment

In addition to genetic evidence, the importance of metabolism to persistence has been supported by the impact of nutrient availability on persister levels. The most comprehensive investigation in this regard explored how the absence of AAs, glucose, ammonium, phosphate, and nucleobases altered persistence to AMP, OFL, and gentamicin (GEN) in *E. coli* (Fung et al., 2010). This study concluded that AA deprivation often increases persistence, mirroring the results from genomic screens that found mutations in AA metabolism to enhance persistence (Table 1). In a study of persister awakening, the number of *E. coli* persisters to AMP and norfloxacin (NOR) were found to be higher when the same stationary-phase culture was inoculated into media unable to support rapid growth resumption (minimal glycerol) in comparison to media with rapid regrowth (LB and minimal glucose) (Joers et al., 2010). Similarly, *E. coli* biofilms have been reported to exhibit higher tolerance to OFL or TIC in fresh media lacking glucose, in comparison to controls with glucose (Bernier et al., 2013). Further support derives from the numerous studies that have shown that nutrient-limited stationary phase and biofilm cultures produce higher persister levels than their exponentially growing counterparts (Spoering and Lewis, 2001; Keren et al., 2004a; Lechner et al., 2012; Bernier et al., 2013). However, it is important to note that high density phenotypes such as quorum signaling may also contribute to persistence in such populations (Möker et al., 2010; Vega et al., 2012).

Taken together, these studies demonstrate that the nutritional environment directly influences persistence, suggesting a central role for metabolism in the persistence phenotype. Further, the mechanisms by which these nutritional stresses enhance persistence have been investigated, and ppGpp has been found to be a key mediator of this process.

## ppGpp, the metabolite controller of persistence

ppGpp and the transcriptional regulator DksA are global regulators of metabolism (Traxler et al., 2006; Dalebroux and Swanson, 2012) that are critical mediators of persistence (Korch et al., 2003; Hansen et al., 2008; Amato et al., 2013; Bokinsky et al., 2013; Germain et al., 2013; Maisonneuve et al., 2013). In *E. coli*, AA limitation stimulates the ribosome-associated RelA to synthesize ppGpp, whereas various stress conditions, such as carbon (Xiao et al., 1991) and fatty acid starvation (Seyfzadeh et al., 1993), stimulate ppGpp synthesis from the cytoplasmic SpoT, which also encodes the sole ppGpp hydrolase. In conjunction with DksA, ppGpp interacts with RNA polymerase to inhibit transcription from stable ribosomal RNA promoters, while simultaneously upregulating transcription of AA biosynthesis and stress response genes (Potrykus and Cashel, 2008; Dalebroux and Swanson, 2012). ppGpp was initially associated with persistence through *hipA7*, a toxin mutant that required ppGpp for its elevated persister phenotype (Korch et al., 2003). Recent work on the native HipA has also shown that its impact on persistence requires ppGpp (Bokinsky et al., 2013; Germain et al., 2013). ppGpp can also increase persistence through its inhibition of exopolyphosphatase (*ppx*), a modulator of the antitoxin degrading Lon protease (Maisonneuve et al., 2013). Additionally, we have demonstrated that RelA, SpoT, and DksA mediate persister formation in response to carbon source transitions (Amato et al., 2013). In particular, we found that the ppGpp biochemical network can act as a metabolic toxin–antitoxin module, where ppGpp is the metabolite toxin and SpoT is its enzymatic antitoxin. We demonstrated that increased ppGpp levels resulted in growth arrest and increased persistence, which could be reverted by SpoT coexpression, and using a mathematical model, we showed that the ppGpp biochemical network can exhibit bistability, where one subpopulation corresponds to normal cells (low ppGpp) and the other to persisters (high ppGpp). Interestingly, RelA–SpoT also demonstrate the prototypical conditional essentiality of a classical toxin–antitoxin system, where the toxin (*relA*) can be deleted, but the antitoxin (*spoT*) can only be removed in a strain without the toxin. In addition to *E. coli*, the stringent response has been shown to impact persistence in other organisms as well. In *P. aeruginosa*, RelA, SpoT, and DksA have all been found to impact persistence (Viducic et al., 2006; Nguyen et al., 2011), whereas in *Mycobacterium tuberculosis*, ppGpp was required for long term survival in an *in vitro* starvation and murine model (Primm et al., 2000; Dahl et al., 2003). Further, the mycobacterial stringent response was shown to exhibit bistability (Ghosh et al., 2011), supporting the assertion that ppGpp is a possible source of phenotypic heterogeneity. In addition, in *Staphylococcus aureus*, ppGpp has been shown to mediate antibiotic tolerance in response to cell envelope stress (Geiger et al., 2014).

These studies demonstrate the importance of the stringent response to persistence and highlight a prevalent mechanism by which metabolic stress can induce persistence. Considering this evidence supporting a central role for ppGpp in persistence, it is attractive to propose that an inhibitor of ppGpp synthesis, such as Relacin (Wexselblatt et al., 2012), or an activator of ppGpp hydrolysis could be effective therapeutics against persisters (Amato et al., 2013; Maisonneuve et al., 2013).

## Persister metabolism as a source of elimination strategies

To date, only a limited number of methods to kill persisters have been discovered, and interestingly, persister metabolism plays a vital role in each approach. For example, the first method, which we co-developed, used metabolites to stimulate proton motive force (pmf) generation in persisters, enabling aminoglycoside (AG) transport and their subsequent killing of *E. coli* and *S. aureus* persisters (Allison et al., 2011b). The participation of persister metabolism was confirmed with genetic mutants and chemical inhibitors, and subsequent studies have found the method to also be effective against *P. aeruginosa* persisters (Barraud et al., 2013). Another method was identified by Kim and colleagues, who screened a chemical library and found that a chemical named C10 promoted fluoroquinolone killing of *E. coli* persisters by stimulating their reversion to a replicating state (Kim et al., 2011). In another study, the quorum-sensing (QS) inhibitor BF8 facilitated elimination of *P. aeruginosa* persisters when combined with CIP or tobramycin (TOB) (Pan et al., 2012). However, upon further analysis, it was discovered that the effect of BF8 was likely due to reactivation of metabolism rather than inhibition of QS. Interestingly, BF8 has also been found to reduce *E. coli* persister levels when combined with OFL, tetracycline (TET), TOB, or GEN (Pan et al., 2013). Recently, another method to eliminate *S. aureus* persisters was discovered by leveraging knowledge that energy levels are low in persisters (Conlon et al., 2013). Specifically, ADEP4, which renders the ClpP protease ATP-independent, led to non-specific protein degradation and death in energy-depleted persisters. Taken together, these studies show that targeting persister metabolism holds great potential for the elimination of these dangerous bacteria and that greater knowledge of persister metabolism will facilitate the discovery of novel therapeutic strategies.

## Methods to measure persister metabolism

Given the potential of persister metabolism to yield anti-persister therapeutics, enhanced metabolic knowledge of these phenotypic variants is desirable. However, direct measurement of metabolites in persisters or assessment of their metabolic activities using conventional approaches, such as mass spectrometry and formazan-based colorimetric assays, are not currently possible due to isolation difficulties (Roostalu et al., 2008; Kint et al., 2012; Orman and Brynildsen, 2013a,b). Although several methods can provide persister-enriched samples (Keren et al., 2004b; Shah et al., 2006), such samples still contain many more other cell-types, such as normal cells and VBNCs, than persisters, and thus are of limited utility for metabolic measurements (Orman and Brynildsen, 2013b). Indeed, the major limitation to segregating persisters from a heterogeneous population is their similarity to VBNCs, which are often more highly abundant. Both VBNCs and persisters stain as live cells, harbor metabolic activity, and are non-growing under antibiotic stress. The only present difference between these subpopulations is that persisters resume growth on standard media, though we note that some VBNCs can regain culturability on non-standard media (Oliver, 2005) suggesting a role for the post-antibiotic environment in defining those cells that survive. Given these technical limitations, we have developed two methods to quantify persister metabolism. The first uses fluorescence activated cell sorting (FACS), a fluorescent measure of metabolic activity, and persistence assays to evaluate the metabolic status of persisters (Orman and Brynildsen, 2013a). This study, which provided the first direct measurement of persister metabolism, demonstrated that *E. coli* persisters largely contained low cellular reductase activity prior to antibiotic stress, confirming previous assumptions about the metabolic activity of the persister state. The second method leveraged the phenomenon by which specific metabolites enabled AG killing of persisters (Allison et al., 2011b). AG potentiation offered a rapid way to measure the breadth of persister metabolic activities (Orman and Brynildsen, 2013b), since the phenomenon relies on persister catabolism of nutrients for pmf generation. Persister metabolic activities are inferred from culturability on standard media, the distinguishing feature between VBNCs and persisters, thereby allowing investigation of persister metabolism even in the presence of VBNCs. This method enabled identification of nutrients metabolized by persisters to different antibiotics (AMP, OFL) during distinct growth stages (exponential, stationary), and thus allowed quantification of heterogeneity in persister metabolism. From these investigations, we demonstrated that glycerol and glucose are the most ubiquitously used carbon sources by various types of persisters, suggesting that the enzymes required for their catabolism are broadly available in persisters.

## Challenges in the study of persister metabolism

The technical hurdles associated with isolation of persisters have hindered understanding of the persister metabolic program and other aspects of persister physiology, including their transcriptome and proteome content. However, FACS offers a technical opportunity to discriminate between VBNC and persister phenotypes. For instance, mixed populations of VBNCs and persisters can be segregated from antibiotic-treated cultures using FACS (Roostalu et al., 2008; Orman and Brynildsen, 2013b), and since VBNCs are much more abundant than persisters in these samples, VBNC physiology can be quantified and potential biomarkers to discriminate between these two cell-types can be found. Nevertheless, any distinguishing features may be condition-specific, since numerous mechanisms can contribute to persister formation (Dhar and McKinney, 2007; Allison et al., 2011a; Balaban, 2011). Indeed, activation of particular pathways will depend on the environment and antibiotic used (Li and Zhang, 2007; Luidalepp et al., 2011), and different formation mechanisms may be active in different growth stages, giving rise to persister heterogeneity, where multiple, distinct persister subpopulations, each with its own unique antibiotic tolerances, coexist in a bacterial culture (Allison et al., 2011a). As a result of heterogeneity, any isolation technique may only capture a fraction of the persisters present, yielding a limited sample of the persister population. Single-cell analysis techniques offer means to interrogate individual cells (Iino et al., 2012, 2013); however, the identification of persisters before they exit their non-replicative state is not presently possible. Perhaps a viable path forward is to study model persisters generated following the over-expression of genes that have been shown to increase persister levels (Korch and Hill, 2006; Vázquez-Laslop et al., 2006). Quantifying metabolic changes in these model systems may provide insight into the physiology and metabolic capabilities of different types of persisters (Bokinsky et al., 2013).

## Conclusion

Persisters embody a medically important bacterial phenotype that relies on metabolism to establish and maintain a dormant, tolerant state during antibiotic stress, and exit that state upon removal of antibiotics (Figure 1). Considerable experimental evidence has accumulated substantiating the importance of metabolism to persistence, and the participation of metabolism in current persister eradication methods provides a convincing argument that enhanced knowledge of the persister metabolic program will accelerate the discovery of additional elimination strategies. However, isolation difficulties impede progress in the understanding of persister physiology, including metabolism. Two potential paths forward are to improve isolation techniques by studying the differences between persisters and VBNCs and to use model persisters to define the breadth and landscape of the persister metabolic program.

### Conflict of interest statement

The authors declare that the research was conducted in the absence of any commercial or financial relationships that could be construed as a potential conflict of interest.
